# 645 Characterization of Rising E-cigarette Burn Injuries from 2013-2022

**DOI:** 10.1093/jbcr/iraf019.274

**Published:** 2025-04-01

**Authors:** Kelsey Glover, Rohit Mittal, Bart Phillips, Steven Kahn

**Affiliations:** South Carolina Burn Center at MUSC; Medical University of South Carolina; BData, Inc; Medical University of South Carolina

## Abstract

**Introduction:**

E-cigarette related burn injuries have been established in literature with extensive documentation of demographics and injury characteristics. With burn repositories now containing more extensive data due to increasing e-cig popularity, investigation into e-cigarette burn injury classification and the impact on the patient healing process is warranted.

**Methods:**

This study was a descriptive review of the 2013-2022 Burn Care Quality Platform (BCQP). The etiology and sub-etiology category was queried for “e-cigarette” with duplicates removed. Demographics, TBSA, length of stay, comorbidities, and type of burn were quiered. Following data collection, all cases were tabulated and analyzed.

**Results:**

From the time-period of 2013-2017 to 2018-2020, there was a 160% relative increase in e-cig burn injuries. The mean TBSA of these burns was 4.5% with a mean LOS of 6.5 days; averaging 1.45 days per 1%TBSA. This is compared to the 1-day per 1% TBSA ratio when averaging the overall data set. When assessed for comorbidities, it was discovered that there is an increased risk of full-thickness burns in obese patients (p< 0.0004), while average LOS was increased in patients with alcoholism (p< 0.033) and diabetes mellitus (p< 0.036).

**Conclusions:**

Patients with comorbidities of DM, obesity, and alcoholism should be warned of increased risk of burn injury if using e-cigs. Furthermore, with a 2.6 fold increase in burn cases over the past ten years, as well as an increased ratio of LOS to TBSA for e-cig burns, further investigation is justified due to increasing healthcare burden of such injuries.

**Applicability of Research to Practice:**

With further research regarding the etiology and outcome of e-cigarette burn injuries, there can be greater advocacy in e-cigarette safety and use in target populations.

**Funding for the Study:**

N/A

